# Knowledge, attitude, and practice regarding radiation exposure and protection among diagnostic radiographers in radiology departments in Shanghai, China

**DOI:** 10.1002/acm2.70559

**Published:** 2026-03-31

**Authors:** Yuanhui Zheng, Die Li, Yongsheng Xiang, Xiaodong Cui

**Affiliations:** ^1^ Department of Medical Imaging Tongji Hospital School of Medicine Tongji University Shanghai China

**Keywords:** attitudes, cross‐sectional study, knowledge, practices, radiation exposure, radiation protection, radiographers

## Abstract

**Background:**

Radiation exposure poses health risks for medical professionals in imaging departments.

**Purpose:**

This study aimed to assess the knowledge, attitudes, and practices (KAP) of diagnostic radiographers regarding radiation exposure and protection.

**Methods:**

A cross‐sectional study was conducted between June and July 2024 among diagnostic radiographers in Shanghai using convenience sampling knowledge (14 items; score range, 0–28), attitudes (9 items; 9–45), and practices (7 items; 9–45).Correlation analysis and structural equation modeling (SEM) explored relationships between KAP domains.

**Results:**

A total of 207 valid questionnaires were collected from diagnostic radiographers. The mean scores were: knowledge 19.05 ± 6.61 (possible range: 0–28), attitude 29.97 ± 5.79 (9–45), and practice 30.51 ± 5.47 (9–45). Correlation analysis revealed significant positive correlations between knowledge‐practice (*r* = 0.390, *p* < 0.001) and attitude‐practice (*r* = 0.143, *p* = 0.039). SEM analysis confirmed knowledge directly affected practice (*β* = 0.446, *p* = 0.006), while attitude did not significantly mediate this relationship.

**Conclusions:**

Radiographers demonstrated insufficient knowledge but moderate attitudes and practices. Based on these findings, tailored education for early‐career radiographers, standardized radiation safety protocols (including clear guidance on protective equipment use), and regular departmental audits may help strengthen radiation safety culture and reduce occupational risks.

## 1 INTRODUCTION

Radiation exposure is a significant health concern, particularly for medical professionals working in environments where radiation is routinely used, such as imaging departments. Studies indicate that prolonged or repeated exposure to low doses of radiation may contribute to adverse health effects, including a potentially heightened risk of cancer, cardiovascular diseases, and other radiation‐induced conditions.^1^, ^2^ Comprehensive reviews have further reinforced these concerns, highlighting that even with current protection standards, healthcare workers with chronic occupational radiation exposure may face increased risks of various malignancies.^3^, ^4^ Recent studies have also demonstrated the impact of occupational radiation exposure on lifetime cancer risk.^5^, ^6^, ^7^ Imaging department radiographers, who are frequently exposed to ionizing radiation during procedures such as X‐rays, CT scans, and fluoroscopy, are particularly vulnerable. As exposure accumulates over the course of a career, concerns about long‐term health effects increase. Documented associations with cataracts and recent research showing increased breast cancer rates among healthcare workers have heightened concerns about the risks of chronic radiation exposure.^8^, ^9^ The long‐term occupational exposure to radiation may significantly raise the risk of cancer, especially in the absence of adequate protection and awareness.^10^ Within this context of radiation risks, radiographers face unique occupational challenges due to their frequent exposure throughout their careers.

Despite these potential risks, there remain significant gaps in the implementation and adherence to radiation protection protocols in medical settings.^11^ Healthcare workers exposed to ionizing radiation are typically equipped with personal dosimeters to monitor their radiation exposure, as mandated by international radiation protection standards such as the International Commission on Radiological Protection (ICRP) Publication 103 and the International Atomic Energy Agency (IAEA) Basic Safety Standards.^12^, ^13^ However, several large‐scale surveys have documented that in practice, not all at‐risk staff consistently utilize these dosimeters, with compliance rates varying from 30% to 85% across different healthcare settings.^14^, ^15^ This variability in adherence to protective measures has been identified as a significant concern in radiation safety programs worldwide. Understanding the gaps in protective practices and the underlying factors influencing healthcare workers’ KAP regarding radiation safety is essential for promoting consistent adherence to safety measures.

To assess the level of radiation safety practices among healthcare workers, the Knowledge, Attitude, and Practices (KAP) survey serves as an important tool, and has been widely validated for radiation safety assessments across different healthcare settings.^16^, ^17^ The KAP model examines a group's understanding, beliefs, and behaviors on a specific issue, particularly in health literacy and public health interventions.

In China, in addition to their professional license, diagnostic radiographers are usually required to obtain the *Radiation Worker Certificate*, which is managed by the medical institution. The *Radiation Worker Certificate* is a qualification that requires specialized training in radiation protection and regular renewal based on health examinations and dose records. This certificate is issued by health administrative departments.^18^ Unlike systems in many Western countries where radiation safety is integrated into professional licensure, China's dual‐credential system creates unique compliance challenges.^18^ Therefore, research specifically targeting Chinese radiographers is necessary to understand the contextual factors affecting radiation safety practices within this regulatory environment.

However, gaps remain in current research regarding the KAP of diagnostic radiographers concerning their own occupational radiation exposure. While several studies have explored radiation safety awareness, large‐scale investigations focusing on radiographers in China are still limited.^19^, ^20^ Despite formal education on radiation protection, specific gaps exist in radiographers' professional practice. The disconnect between theoretical knowledge and clinical application,^21^ insufficient emphasis on occupational self‐protection in Chinese undergraduate curricula,^22^ and inadequate evaluation in continuing education programs^23^ all highlight the need for targeted KAP assessment. Therefore, this study aims to address these gaps by providing insights into the KAP of diagnostic radiographers.

## 2 MATERIALS AND METHODS

### 2.1 Study design and subjects

This cross‐sectional study was conducted in medical institutions between June and July 2024, with diagnostic radiographers as the study participants. The study was approved by the Ethics Committee of our Hospital, and informed consent was obtained from all participants. Informed consent was obtained electronically on the first page of the questionnaire, which clearly explained the study's purpose, voluntary participation, and data confidentiality prior to beginning the survey. Responses were collected anonymously, and no personally identifying information was requested in the questionnaire. The inclusion criteria were diagnostic radiographers working in radiology departments. Exclusion criteria included: (1) doctors involved in radiological work but not directly exposed to radiation, as they typically do not perform imaging procedures and thus face different occupational exposure profiles; as well as women who were currently pregnant or on maternity leave; (2) individuals under 18 years of age; and (3) respondents whose questionnaire responses were incomplete (i.e., partial submissions with unanswered items) or illogical (i.e., internally inconsistent answers on built‐in duplicate checks). For example, the demographic section contained a duplicated “professional title” item as an attention/trap check; responses were considered illogical and excluded if the two answers did not match.

Convenience sampling was used. Questionnaires were distributed through radiology‐related department communities, members of the Imaging Technology Division of the Shanghai Medical Association, and WeChat groups. Responses were collected from the Radiology Departments of several public tertiary hospitals, including Shanghai Tongji Hospital, Shanghai Pulmonary Hospital, Shanghai Chest Hospital, Shanghai Zhongshan Hospital, Shanghai East Hospital, Shanghai Renji Hospital, Shanghai Huashan Hospital, Shanghai Tongren Hospital, and Shanghai Changzheng Hospital, as well as private hospitals such as Shanghai Yaoying Hospital and Shanghai Jiahui International Hospital. The electronic survey was created using the Wenjuanxing platform, generating a link that was shared with participants via WeChat or QQ. Respondents could access the questionnaire by clicking the link, and to ensure data quality, each IP address was allowed to submit only once, with all questions marked as mandatory. All hospitals were located in urban areas, and the majority were public tertiary institutions. The “other” category in our analysis included secondary hospitals, primary hospitals, and private institutions. To ensure data quality, IP address restrictions were implemented to prevent duplicate entries. In addition, members of the research team conducted a detailed review of the collected data to assess completeness and logical consistency. To ensure that the survey reached only the intended participants, the link was distributed exclusively through internal communication channels within radiology departments, professional radiology associations, and closed WeChat groups consisting solely of diagnostic radiographers. During questionnaire distribution, participants were encouraged to complete the survey independently. In particular, they were reminded not to consult external materials (e.g., websites or guidelines) or use generative AI tools when answering the knowledge items. Responses were reviewed for logical consistency by the research team. Responses with incomplete answers to mandatory questions were excluded. Logical inconsistencies that led to exclusion included: outliers in demographic data (e.g., implausibly low or high age values), contradictory symptom reporting (e.g., selecting both specific health symptoms and “none of the above”), and inconsistent responses to pre‐set duplicate questions that were designed to detect random answering patterns.

### 2.2 Questionnaire

The questionnaire design was informed by references to relevant literature,^24^, ^25^ as well as the “*Expert Consensus on Occupational Health Surveillance of Radiation Workers*” by the Branch of Nuclear Emergency Medicine, Chinese Nuclear Society. After the initial design, it was refined based on feedback from several senior experts and tested in a small‐scale pilot study (51 respondents), resulting in a reliability score of 0.760.

The final version of the questionnaire, written in Chinese, covered four main aspects: demographic information (age, gender, marital status, children, education level, professional title, years of work experience, possession of “Radiation Worker Certificate,” average daily imaging examinations performed, hospital grade, experience of health conditions or symptoms, perception of health conditions related to radiation exposure), knowledge, attitudes, and practices. The knowledge dimension comprised 14 questions, with questions 1, 10, and 11 assessing the respondents' understanding of key concepts. These items were further classified into conceptual questions (e.g., understanding of radiation definitions, health surveillance) and factual questions (e.g., radiation limits, symbol recognition, clinical safety protocols), allowing more nuanced assessment of knowledge domains. Responses to these were scored as “understand” (2 points), “partially understand” (1 point), and “do not understand” (0 points). For the remaining knowledge questions, correct answers were awarded 2 points, while incorrect or unclear answers were given 0 points, yielding a total score range of 0–28 points for this section. The attitude dimension included nine questions, measured on a Likert scale, with reverse scoring applied for questions 4–9, which represented negative attitudes. Responses ranged from “strongly agree” (5 points) to “strongly disagree” (1 point), resulting in a total score range of 9–45 points. Finally, the practice dimension had seven questions, also using a Likert scale, with responses ranging from “always” (5 points) to “never” (1 point), with a total score range of 9–45 points. Participants who scored above 80% of the total were categorized as having adequate knowledge, positive attitude, and proactive practice, based on Bloom's cutoff. Those falling within the range of 60%–80% of the total were classified as having moderate knowledge, attitude, and practice. Scores below 60% of the total were indicative of inadequate knowledge, negative attitude, and inactive practice, in alignment with Bloom's classification criteria.^26^

Construct validity of the questionnaire was supported by confirmatory factor analysis (CFA), with acceptable model fit indices (RMSEA = 0.053, SRMR = 0.070, TLI = 0.909, CFI = 0.921). The CFA was conducted separately for the three domains—knowledge, attitude, and practice—to account for the different scoring formats and latent structures. Each domain demonstrated acceptable model fit, supporting the construct validity of the respective scales. The Kaiser–Meyer–Olkin (KMO) measure was 0.843, indicating sampling adequacy for factor analysis. Although a formal content validity index (CVI) was not originally calculated, we have since estimated the average expert agreement level during item evaluation. Based on assessments by four senior radiology and occupational health experts, the item‐level agreement exceeded 0.80, indicating acceptable content validity. Face validity was evaluated during the pilot test, in which 51 participants assessed item clarity and relevance. Questionnaire items were designed based on Chinese national radiation protection standards and training materials, reflecting the knowledge framework within which participants are trained and evaluated.

### 2.3 Statistical analysis

Data analysis was conducted using SPSS 27.0 and AMOS 26.0 (IBM, Armonk, NY, USA). Descriptive statistics were performed on the demographic information and KAP scores, with continuous data presented as means and standard deviations (SD), while categorical data were expressed as *n* (%). The normality of continuous variables was assessed using the Kolmogorov–Smirnov test, and homogeneity of variance was evaluated using Levene's test. To compare Knowledge (K), Attitude (A), and Practice (P) scores across different demographic groups, the *t*‐test was employed for two‐group comparisons, while ANOVA was used for comparisons involving three or more groups. For multiple comparisons following ANOVA, Bonferroni post‐hoc tests were performed. Effect sizes were calculated using Cohen's *d* for *t*‐tests and *η* ^2^ for ANOVA to quantify the magnitude of group differences. These values were then converted to correlation coefficient *r* to provide a standardized measure of effect size. The magnitude of *r* was interpreted as: small effect (0.1 ≤ *r* < 0.3), moderate effect (0.3 ≤ *r* < 0.5), and large effect (*r* ≥ 0.5). Structural equation modeling (SEM) was applied to explore the interaction among KAP. SEM was chosen due to its capacity to evaluate complex relationships between latent variables—knowledge, attitudes, and practices—simultaneously. This approach allows for the testing of both direct and indirect effects, offering a more comprehensive assessment of the KAP theoretical framework than traditional regression methods. A two‐sided *p* < 0.05 was value statistically significant.

To further validate findings from SEM, additional path analysis was conducted using total KAP scores without considering individual items. This simplified model provided a more direct examination of the relationships between the three domains. Additionally, regression analysis was performed to assess factors associated with good knowledge, attitude, and practice, with binary outcome variables created based on Bloom's cutoff points. For regression analysis, variables were categorized as adequate (scores ≥ 80% of maximum) versus inadequate/moderate (scores < 80% of maximum). Adjusted odds ratios (OR) with 95% confidence intervals were calculated after controlling for demographic variables. For all analyses, statistical significance was set at *p* < 0.05.

## 3 RESULTS

### 3.1 Demographic characteristics on participants

A total of 252 questionnaires were initially collected, and 45 were excluded due to incomplete responses (*n* = 23) or logical errors (*n* = 22). These logical errors included one outlier in reported age (e.g., implausibly low or high value), four cases in which participants selected both specific health symptoms and “none of the above,” and 17 cases with contradictory answers to pre‐set duplicate questions designed to detect inconsistencies. This resulted in 207 valid questionnaires. Of these, 129 participants (62.32%) were 30 years old or younger, 122 (58.94%) were female, 147 (71.01%) had a Bachelor's degree, 155 (74.88%) held a Radiation Worker Certificate. Among the participants, 104 (50.24%) experienced health issues, of which hair loss (77 [37.20%]) and insomnia 72 ([34.78%]) were most common. And 76 (36.71%) participants believed their health issues were related to radiation exposure at work (Table 1).

**TABLE 1:** Demographic characteristics of participants.

Characteristics	*N* (%)
Total	207
Age, years	
≤30	129 (62.32)
>30	78 (37.68)
Gender	
Male	85 (41.06)
Female	122 (58.94)
Marital status	
Married	97 (46.86)
Other	110 (53.14)
Children	
Yes	75 (36.23)
No	132 (63.77)
Education	
College or below	35 (16.91)
Bachelor's degree	147 (71.01)
Master's degree or above	25 (12.08)
Professional title	
Junior	92 (44.44)
Intermediate	65 (31.40)
Senior	6 (2.90)
No title	44 (21.26)
Work experience (years)	
≤5	98 (47.34)
>5	109 (52.66)
Radiation worker certificate	
Yes	155 (74.88)
No	52 (25.12)
Average daily imaging examinations	
10–50	91 (43.96)
50–100	44 (21.26)
100–200	46 (22.22)
200 or above	26 (12.56)
Hospital grade	
Tertiary Hospital	134 (64.73)
Others (including secondary hospitals, primary hospitals, and private institutions)	73 (35.27)
Health conditions or symptoms (can choose multiple answers)	
Hair loss	77 (37.20)
Insomnia	72 (34.78)
Decreased white blood cells found in medical examination	14 (6.76)
Endocrine abnormalities found in medical examination	13 (6.28)
Thyroid nodules	32 (15.46)
None of the above	93 (44.92)
Perceived radiation‐related health issues	
Yes	76 (36.71)
No	38 (18.36)

### 3.2 Knowledge, attitude, and practice

The mean scores were 19.05 ± 6.61 for knowledge (range: 0–28), 29.97 ± 5.79 for attitude, and 30.51 ± 5.47 for practice (both range: 9–45). According to Bloom's classification, the knowledge mean score falls within the “moderate” level (60%–80% of the maximum score), but is close to the threshold for “inadequate” knowledge (<60%). The attitude and practice scores are also in the “moderate” range. These findings suggest that while diagnostic radiographers have a fair understanding and engagement in protective behaviors, their overall knowledge is still insufficient and requires improvement. In terms of categorical distribution based on Bloom's cutoff, 45 participants (21.74%) had inadequate knowledge, 98 (47.34%) had moderate knowledge, and 64 (30.92%) had adequate knowledge. For attitude, 39 participants (18.84%) were classified as having negative attitudes, 114 (55.07%) had moderate attitudes, and 54 (26.09%) had positive attitudes. Regarding practice, 47 participants (22.71%) demonstrated inactive practices, 84 (40.58%) had moderate practices, and 76 (36.71%) exhibited proactive practices. Analysis of demographic characteristics showed significant differences in knowledge and practice scores across education level (*p* < 0.001 and *p* < 0.001), job grade (*p* < 0.001 and *p* < 0.001), years of work experience (*p* < 0.001 and *p* < 0.001), Radiation Worker Certificate status (*p* < 0.001 and *p* < 0.001), and job title (*p* < 0.001 and *p* < 0.001). Additionally, participants over 30 years old (*p* < 0.001, *p* = 0.003, and *p* = 0.004) and those working in tertiary hospitals (*p* = 0.001, *p* = 0.009, and *p* < 0.001) had higher knowledge, attitude, and practice scores. Attitude scores were higher among participants with fewer daily imaging examinations (*p* = 0.009) and those who did not attribute their condition to radiation exposure (*p* < 0.001). Participants who were married (*p* = 0.033) and had children (*p* = 0.045) were more likely to have higher practice scores. Specifically, further analysis revealed an interesting disconnect between radiation knowledge and health perceptions. Among those who attributed their health issues to radiation exposure (*n* = 76), their knowledge scores (20.05 ± 4.92) were not significantly different from those who did not make this attribution (19.08 ± 6.67, *p* = 0.380). However, participants who believed their health issues were related to radiation exposure showed significantly lower attitude scores (27.76 ± 4.63 vs. 31.37 ± 4.36, *p* < 0.001), indicating that health concerns may influence attitudes more strongly than knowledge levels (Table 2).

**TABLE 2:** Knowledge, attitude, and practice scores by demographic characteristics.

	Knowledge			Attitude			Practice		
	Mean ± SD	*p*	*r*	Mean ± SD	*p*	*r*	Mean ± SD	*p*	*r*
Total									
Age, years		<0.001	0.897		0.003	0.635		0.004	0.590
≤30	17.81 ± 7.27			29.04 ± 5.73			29.67 ± 5.94		
>30	21.12 ± 4.69			31.50 ± 5.59			31.91 ± 4.26		
Gender		0.197	0.117		0.810	0.004		0.807	0.004
Male	19.76 ± 6.82			30.08 ± 5.71			30.62 ± 5.62		
Female	18.56 ± 6.43			29.89 ± 5.87			30.43 ± 5.38		
Marital status		0.055	0.259		0.929	0.001		0.033	0.319
Married	19.99 ± 6.36			29.93 ± 6.01			31.37 ± 5.04		
Other	18.23 ± 6.73			30.00 ± 5.62			29.75 ± 5.73		
Children		0.066	0.238		0.627	0.017		0.045	0.282
Yes	20.17 ± 6.44			30.23 ± 6.25			31.52 ± 4.76		
No	18.42 ± 6.64			29.82 ± 5.53			29.94 ± 5.77		
Education		<0.001	0.506		0.831	0.013		<0.001	0.644
College or below	15.29 ± 8.69			29.83 ± 5.80			27.06 ± 7.17		
Bachelor's degree	19.85 ± 5.98			30.10 ± 5.84			31.12 ± 4.82		
Master's degree or above	19.64 ± 5.00			29.36 ± 5.67			31.80 ± 4.55		
Professional title		<0.001	1.624		0.050	0.185		<0.001	0.580
Junior	19.99 ± 5.47			29.12 ± 5.89			30.57 ± 5.27		
Intermediate	21.55 ± 3.65			30.42 ± 5.42			32.06 ± 4.14		
Senior	23.33 ± 2.07			26.50 ± 7.84			35.00		
No title	12.82 ± 8.46			31.55 ± 5.50			27.5 ± 6.56		
Work experience (years)		<0.001	1.565		0.052	0.265		<0.001	1.321
≤5	16.87 ± 7.72			29.14 ± 5.59			28.84 ± 6.25		
>5	21.02 ± 4.63			30.71 ± 5.89			32.02 ± 4.13		
Radiation worker certificate		<0.001	4.297		0.359	0.059		<0.001	1.719
Yes	20.89 ± 4.38			30.18 ± 5.89			31.55 ± 4.55		
No	13.58 ± 8.80			29.33 ± 5.50			27.42 ± 6.72		
Average daily imaging examinations		0.150	0.124		0.009	0.277		0.419	0.066
10–50	17.90 ± 7.34			31.15 ± 5.87			29.90 ± 6.27		
50–100	19.61 ± 6.28			29.59 ± 5.32			30.41 ± 5.54		
100–200	19.91 ± 6.09			29.72 ± 5.48			31.39 ± 4.32		
200 or above	20.62 ± 4.64			26.88 ± 5.82			31.27 ± 3.84		
Hospital grade		0.001	0.830		0.009	0.484		<0.001	0.988
Tertiary hospital	20.19 ± 5.27			30.74 ± 6.04			31.54 ± 4.78		
Other	16.96 ± 8.17			28.55 ± 5.04			28.63 ± 6.15		
Health conditions or symptoms (can choose multiple answers)									
Hair loss	19.57 ± 5.43	0.965	−0.003	28.51 ± 4.88	0.965	0.202	30.71 ± 4.70	0.965	0.039
Insomnia	19.26 ± 5.77	0.783	0.019	27.86 ± 4.46	0.783	0.278	28.88 ± 5.69	0.783	0.244
Decreased white blood cells found in medical examination	19.21 ± 6.61	0.985	0.001	28.79 ± 6.18	0.985	0.057	28.29 ± 7.15	0.985	0.085
Endocrine abnormalities found in medical examination	20.38 ± 4.01	0.816	−0.016	27.62 ± 5.01	0.816	0.107	30.85 ± 3.78	0.816	0.036
Thyroid nodules	21.28 ± 3.21	0.1477	−0.101	28.59 ± 4.58	0.1477	0.096	31.38 ± 4.32	0.1477	−0.026
None of the above	18.23 ± 7.65	0.410	0.057	31.19 ± 6.61	0.410	−0.199	30.80 ± 5.66	0.410	−0.096
Perceived radiation‐related health issues		0.380	0.054		<0.001	1.110		0.805	0.004
Yes	20.05 ± 4.92			27.76 ± 4.63			30.37 ± 5.18		
No	19.08 ± 6.67			31.37 ± 4.36			30.11 ± 5.64		

*Note*: Post‐hoc Bonferroni tests revealed significant differences between: Bachelor's versus College or below (Knowledge: *p* = 0.001; Practice: *p* < 0.001), Master's versus College or below (Knowledge: *p* = 0.031; Practice: *p* = 0.002). Effect sizes (*r*) are presented to quantify the magnitude of group differences: small effect (0.1 ≤ *r* < 0.3), moderate effect (0.3 ≤ *r* < 0.5), and large effect (*r* ≥ 0.5)

**FIGURE 1:**
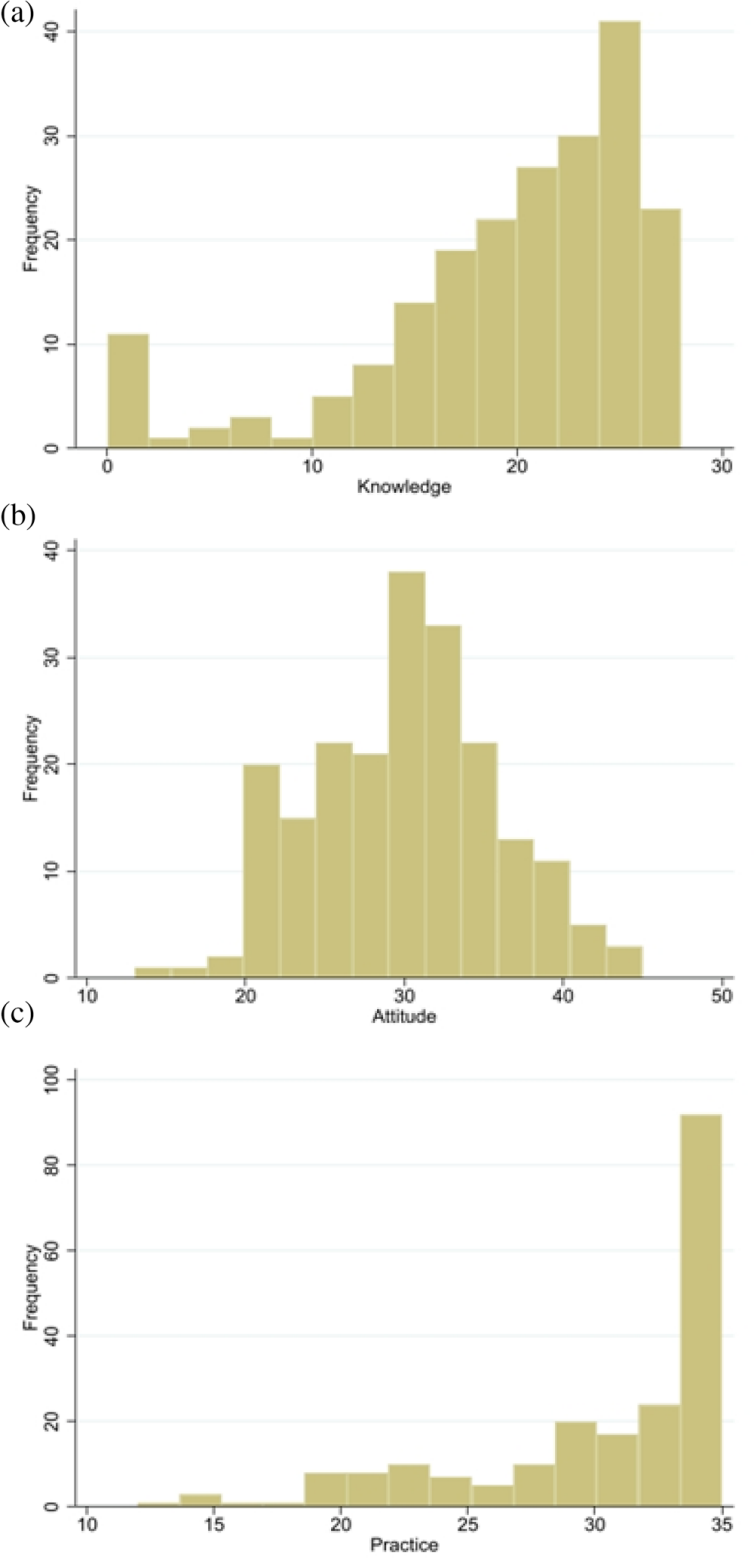
Distribution of knowledge, attitude, and practice scores among diagnostic radiographers (*n* = 207). (a) Distribution of knowledge scores, (b) distribution of attitude scores, and (c) distribution of practice scores.

The distribution of Knowledge, Attitude, and Practice scores is presented in Figure 1. The Knowledge scores show a bimodal distribution, with peaks around 15 and 22 points. Attitude scores present a more normal distribution centered around 30 points, while Practice scores show a slightly positively skewed distribution with the majority of participants scoring between 28 and 35 points.

In the knowledge dimension, 23.19% of participants reported not understanding the concept of “health surveillance” (K3). The three knowledge items with the lowest accuracy rates were: “CT scans (in separate rooms) do not require personal protective equipment and auxiliary protective facilities for radiological workers” (K9) with 21.74%, “The meaning of the following symbol is to beware of ionizing radiation” (K8) with 39.61%, and “The annual dose equivalent limit for other individual organs or tissues is 500 mSv” (K6) with 58.94% (Table S1).

Regarding attitudes, 32.37% strongly agreed, and 19.81% agreed that they were overworked (A4), 14.98% strongly agreed, and 14.49% agreed they were exposed to excessive radiation at work (A5), 18.36% strongly agreed, and 17.87% agreed that their health had been affected by radiation (A6), and 22.71% strongly agreed, and 25.12% agreed that they were very concerned about potential occupational exposure (A8).

In the practice dimension, 6.28% occasionally and 4.35% never wore protective equipment as required (P5), 4.35% occasionally and 4.83% never wore personal dosimeters (P4), and 3.86% occasionally and 3.86% never underwent “Occupational health checks for radiological workers” (P2) (Table S2).

### 3.3 Interaction among KAP

Correlation analysis revealed varying degrees of associations among KAP components. A moderate positive correlation was observed between knowledge and practice scores (*r* = 0.390, *p* < 0.001). Although statistically significant, only a weak correlation was found between attitude and practice scores (*r* = 0.143, *p* = 0.039). Notably, there was no significant correlation between knowledge and attitude scores (*r* = 0.120, *p* = 0.084) (Table S3). These findings indicate that the relationships between KAP components are more complex than typically assumed in occupational health interventions.

SEM analysis indicated that knowledge had a direct effect on practice (*β* = 0.446, 95% CI: 0.258–0.674, *p* = 0.006). An additional mediating effect analysis was conducted based on the study hypothesis, revealing that neither the effect of knowledge on attitude (*β* = 0.100, 95% CI: –0.058–0.264, *p* = 0.168) nor the effect of attitude on practice (*β* = –0.017, 95% CI: –0.192–0.122, *p* = 0.793) was statistically significant. The indirect effect of knowledge on practice through attitude was also not significant (*β* = –0.002, 95% CI: –0.046–0.013, *p* = 0.641). The model demonstrated good fit to the data (CMIN/DF = 1.887, RMSEA = 0.066, IFI = 0.876, TLI = 0.862, CFI = 0.874), indicating the reliability of these pathway estimates (Tables 3 and S4).

**TABLE 3:** SEM analysis.

			*β*	*p*
Attitude	<—	Knowledge	0.173	0.205
Practice	<—	Attitude	−0.017	0.811
Practice	<—	Knowledge	0.757	<0.001
K1	<—	Knowledge	0.710	<0.001
K2	<—	Knowledge	0.826	<0.001
K3	<—	Knowledge	0.855	<0.001
K4	<—	Knowledge	0.922	<0.001
K5	<—	Knowledge	1.000	
K6	<—	Knowledge	0.893	<0.001
K7	<—	Knowledge	0.884	<0.001
K8	<—	Knowledge	0.371	0.005
K9	<—	Knowledge	0.266	0.017
K10	<—	Knowledge	0.782	<0.001
K11	<—	Knowledge	0.706	<0.001
K12	<—	Knowledge	0.868	<0.001
K13	<—	Knowledge	0.777	<0.001
K14	<—	Knowledge	0.786	<0.001
A9	<—	Attitude	0.658	<0.001
A8	<—	Attitude	0.900	<0.001
A7	<—	Attitude	0.877	<0.001
A6	<—	Attitude	1.000	
A5	<—	Attitude	0.942	<0.001
A4	<—	Attitude	0.766	<0.001
A3	<—	Attitude	0.017	0.809
A2	<—	Attitude	0.040	0.448
A1	<—	Attitude	0.030	0.590
P1	<—	Practice	0.831	<0.001
P2	<—	Practice	1.000	
P3	<—	Practice	0.926	<0.001
P4	<—	Practice	0.959	<0.001
P5	<—	Practice	0.575	<0.001
P6	<—	Practice	0.471	<0.001
P7	<—	Practice	0.474	<0.001

To further validate these findings, we conducted additional path analysis focusing on the total KAP scores without considering individual items. This simplified model confirmed our SEM results: knowledge had a significant direct effect on practice (*β* = 0.312, 95% CI: 0.208–0.416, *p* < 0.001), while neither the effect of knowledge on attitude (*β* = 0.105, 95% CI: –0.012–0.224, *p* = 0.081) nor attitude on practice (*β* = 0.092, 95% CI: –0.026–0.211, *p* = 0.127) reached statistical significance (Table S5 and Figure S1). The indirect effect of knowledge on practice through attitude remained non‐significant (*β* = 0.009, 95% CI: –0.006–0.026, *p* = 0.25). Additionally, regression analysis revealed that after adjusting for demographic factors, both knowledge (OR = 1.177, 95% CI: 1.087–1.275, *p* < 0.001) and attitude (OR = 1.115, 95% CI: 1.030–1.208, *p* = 0.007) were independently associated with good practice (Tables S6 and S7).

## 4 DISCUSSION

Diagnostic radiographers demonstrated insufficient knowledge but moderate attitudes and practices concerning radiation exposure and protection. Targeted educational interventions focusing on improving radiographers' knowledge of radiation safety may enhance their protective practices, ultimately reducing the potential health risks associated with occupational exposure.

The correlation analyses and SEM results together showed that knowledge had a moderate positive correlation with practice and a direct effect on practice, suggesting that improving knowledge could enhance protective practices. The weak correlation between attitude and practice, despite being statistically significant, suggests that other factors may mediate this relationship. Most notably, the lack of a significant correlation between knowledge and attitude, together with the absence of a mediating role of attitude, challenges the conventional KAP model, which assumes that knowledge influences behavior through changes in attitude.^27^, ^28^ One possible explanation is that radiographers' practices may be shaped more by institutional enforcement (e.g., routine checks, mandatory training, and protective protocols) than by personal attitudes alone. Prior studies similarly report that higher knowledge is associated with better adherence to safety measures^29^, ^30^ having sufficient knowledge may influence protective behaviors through institutional enforcement mechanisms, even when individual attitudes are less favorable. Studies from Japan^25^ and the United States^29^ found that organizational protocols and department‐level safety programs more effectively ensured compliance than individual attitudes alone, suggesting that institutional frameworks play a crucial role in translating knowledge into consistent protective behaviors. Notably, while the direct pathway from attitude to practice was not significant in our structural models, regression analysis revealed that attitude remains an independent predictor of good practice, suggesting a more complex relationship than the traditional linear KAP model implies. This discrepancy may reflect differences in analytical approaches and the moderate attitude levels observed, where knowledge may not fully translate into improved attitudes.

The significant differences in KAP based on variables such as age, education, job grade, and years of work experience warrant detailed discussion. Older radiographers and those with more work experience had higher knowledge and practice scores, possibly due to more exposure to training and workplace policies.^31^, ^32^ In contrast, younger and less experienced workers exhibited lower scores, suggesting a need for targeted interventions for this group. Education level was another key factor, with those holding a Bachelor's or higher degree performing better in both knowledge and practice. This reinforces the importance of formal education in equipping radiographers with necessary radiation safety skills. Undergraduate programs should strengthen modules on radiation biology and protection principles, while continuing education should be tailored to educational background, with more intensive interventions for those with lower formal education.^21^, ^33^ Educational institutions could develop specialized certifications in radiation safety that bridge theoretical knowledge and practical implementation, focusing particularly on identified knowledge gaps such as CT protection requirements.^34^ Interestingly, no significant differences in attitude were found across these variables, suggesting that attitude may be more resistant to change compared to knowledge and practice, which could be a result of ingrained workplace culture.

The results showed several areas of insufficient knowledge, particularly in understanding ionizing radiation, health surveillance, and radiation protection measures. For example, a large proportion of radiographers did not understand the use of protective equipment for CT scans, which contrasts with findings from other studies showing better knowledge in similar settings.^35^, ^36^ These gaps support focused refresher training on identified weak items, with emphasis on correct use of personal protective equipment.^37^, ^38^

The attitudes toward radiation protection were generally moderate, but certain areas showed concerning trends. For example, a significant number of radiographers expressed concerns about excessive radiation exposure and occupational risks, which may reflect underlying anxieties about workplace safety. These concerns are similar to findings in other studies where healthcare workers report being overworked and under‐protected.^39^, ^40^ Our findings revealed a notable paradox: despite relatively adequate knowledge levels, 36.71% of participants attributed their health issues to radiation exposure, with no significant difference in knowledge scores between those who did and did not make this attribution. This suggests that technical knowledge alone may not be sufficient to address workplace concerns about radiation exposure. Clear communication from management about the actual risks of radiation exposure, along with regular health monitoring and feedback, may help bridge this gap between knowledge and health risk perception. Additionally, the provision of adequate resources and enhanced protective measures could help alleviate these concerns.^41^, ^42^

These practice gaps align with the compliance issues documented in the literature and may be attributed to several barriers. Our data indicated that 32.37% of participants strongly agreed they were overworked, which likely contributes to these observed adherence gaps.^15^ Institutional factors also play a crucial role; inadequate availability of protective equipment, unclear departmental policies, or inconsistent enforcement of safety regulations can undermine even knowledgeable radiographers' motivation to follow protection guidelines.^43^ Additionally, an organizational culture that does not prioritize radiation safety may further compromise adherence. The finding that radiographers in tertiary hospitals demonstrated better practices suggests that robust institutional infrastructure and support systems are essential for promoting radiation safety. For instance, some radiographers reported never wearing dosimeters or protective gear, which contrasts with findings from studies reporting high compliance with safety measures.^44^ To improve practices, strict enforcement of protective policies is needed, alongside regular audits to ensure compliance. Additionally, increasing access to personal protective equipment and fostering a safety culture through role modeling and continuous education can further enhance adherence to protective practices. Specific attention should be given to junior radiographers and those without formal titles, who demonstrated the poorest practices.^45^, ^46^

Compare with similar KAP studies previously conducted in other countries, this study revealed both similarities and differences in radiation protection knowledge, attitudes, and practices among healthcare workers in different healthcare systems (Table 4). For instance, Yashima and Chida's study from Japan supported the importance of organizational protocols on radiation protection compliance.^25^ Alkhayal et al.’s study in Saudi Arabia identified similar knowledge gaps with our study, particularly regarding radiation protection measures.^24^ The study by Yurt et al. in Turkey showed higher knowledge scores but similar challenges in translating this knowledge into consistent protective practices.^20^ These comparative insights highlight common challenges in radiation safety education and implementation across different regional contexts.

**TABLE 4:** Comparison of findings from different countries in Asia.

Countries	Population	Date	Duration	Main finding
China (current study)	Diagnostic radiographers	2024	June–July 2024	Knowledge score: 19.05 ± 6.61; direct correlation between knowledge and practice (*β* = 0.446)
Japan	Medical radiographers	2022	Not specified	Organizational protocols had stronger impact than personal attitudes on radiation protection
Saudi Arabia	Healthcare workers in tertiary center	2023	Not specified	Moderate knowledge levels with gaps in radiation protection measures
Turkey	Dentists	2022	Not specified	Knowledge score: 75.6%; moderate correlation between knowledge and practice (*r* = 0.31)

This study makes several unique contributions to the field of radiation protection in China. First, while previous studies have examined radiation safety awareness among general healthcare workers, this is one of the first large‐scale investigations focusing exclusively on diagnostic radiographers in Chinese hospitals. The findings provide crucial baseline data for this specific group. Second, our analysis reveals important disparities across different professional ranks and experience levels, which has not been previously documented in the Chinese healthcare context. Third, by examining the relationships between knowledge, attitudes, and practices, we provide novel insights into how these factors interact in the Chinese healthcare system, where radiation safety training is regulated through a unique dual‐credential system. These findings have important implications for policy development, suggesting that radiation safety programs in China should consider both individual factors (such as education level and years of experience) and institutional factors (such as hospital grade and departmental protocols) when designing interventions. Future research could build on these findings by examining the effectiveness of targeted interventions for specific subgroups or investigating how the dual‐credential system influences radiation safety practices compared to integrated systems in other countries.

This study has several important limitations. First, its cross‐sectional design limits the ability to infer causal relationships between knowledge, attitudes, and practices. Second, all variables were self‐reported, which may introduce bias, particularly for sensitive items. Although the survey was anonymous, attitude and practice responses may still be affected by social desirability bias. Additionally, online completion in an uncontrolled setting could not fully exclude inappropriate assistance (e.g., consulting external resources or using generative AI tools), which may have biased knowledge assessment. Third, the study was conducted exclusively in Shanghai urban hospitals, limiting generalizability to other regions with different healthcare infrastructure and safety standards. Fourth, our questionnaire was developed based on Chinese radiation protection standards and training curricula in early 2024. Some items (e.g., dose‐limit wording and CT‐related protection practices) may differ from recent international guidelines (ICRP Publication 103 and related updates);^47^, ^48^ nevertheless, the instrument reflects the knowledge framework used for training and evaluation in China. Fifth, contextual confounders such as departmental safety policies, workload, and equipment availability were not captured and may have influenced KAP scores. Future studies should consider correlating self‐reported data with objective indicators such as dosimetry readings or medical records to improve the strength of causal inferences.

## 5 CONCLUSIONS

In conclusion, medical diagnostic radiographers demonstrated gaps in knowledge, along with moderate attitudes and practices concerning radiation exposure and protection. Our findings highlight the need for targeted educational interventions focused on junior staff, less experienced radiographers, and those with lower educational attainment. Specific knowledge gaps—such as understanding ionizing radiation symbols (39.61% accuracy) and CT scan protection requirements (21.74% accuracy)—should be prioritized in future training. Differentiated strategies—such as mandatory workshops, online training modules, and hands‐on practical sessions—should be tailored to different levels of experience and professional titles to enhance the effectiveness of radiation safety education.

## AUTHOR CONTRIBUTIONS

Yuanhui Zheng carried out the studies, participated in collecting data, and drafted the manuscript. Die Li, Yongsheng Xiang, and Xiaodong Cui performed the statistical analysis and participated in its design. Yuanhui Zheng participated in acquisition, analysis, or interpretation of data and draft the manuscript. All authors read and approved the final manuscript.

## ETHICS STATEMENT

This work has been carried out in accordance with the Declaration of Helsinki (2000) of the World Medical Association. The study was approved by the Ethics Committee of Shanghai Tongji Hospital (K‐W‐2024‐005), and informed consent was obtained from all participants.

## CONFLICT OF INTEREST STATEMENT

The authors declare that they have no competing interests.

## Supporting information




**Supporting File 1**: acm270559‐sup‐0001‐SuppMat.docx.

**Supporting File 2**: acm270559‐sup‐0002‐Figure‐S01.tif.

## Data Availability

Researchers with an interest can access the data by contacting the first author.
