# Supplementary material for: Knowledge, attitude, and practice regarding radiation exposure and protection among diagnostic radiographers in radiology departments in Shanghai, China
Source: J Appl Clin Med Phys. 2026 Mar 31;27(4):e70559. doi: 10.1002/acm2.70559 (PMC13140916; doi:10.1002/acm2.70559)
Supplement: Supplementary file 1 — Supporting File 1: acm270559‐sup‐0001‐SuppMat.docx. [file ACM2-27-e70559-s002.docx]

**Supplementary Materials**

Supplementary Figure 1: Path analysis of the relationships between total Knowledge, Attitude, and Practice scores.

Supplementary Table 1. Knowledge responses among diagnostic radiographers

| **Knowledge** | **Understand N**(%) | **Partially Understand N**(%) | **Do not understand N**(%) |
| --- | --- | --- | --- |
| **1. Do you understand the definition of ionizing radiation?** | 135 (65.22) | 60 (28.99) | 12 (5.80) |
| **10. Do you understand the methods of handling occupational radiation exposure?** | 103 (49.76) | 79 (38.16) | 25 (12.08) |
| **11. Do you understand the concept of "health surveillance"?** | 78 (37.68) | 81 (39.13) | 48 (23.19) |
| **Knowledge** |  | **Correct N**(%) |  |
| **2. Radiation-induced injuries include deterministic effects and stochastic effects, with stochastic effects potentially caused by lower doses of radiation.** |  | 133 (64.25) |  |
| **3. The most radiation-sensitive parts of the body are the gonads and hematopoietic organs.** |  | 177 (85.51) |  |
| **4. For personnel engaged in radiological work, the annual dose equivalent limit for whole-body uniform exposure is 50 mSv.** |  | 162 (78.26) |  |
| **5. For personnel engaged in radiological work, the annual dose equivalent limit for the eyes is 150 mSv.** |  | 134 (64.73) |  |
| **6. For other individual organs or tissues, the annual dose equivalent limit is 500 mSv.** |  | 122 (58.94) |  |
| **7. Excessive radiation exposure may cause symptoms such as nausea, vomiting, bleeding, skin peeling, insomnia, hair loss, and decreased immunity.** |  | 182 (87.92) |  |
| **8. The meaning of the following symbol is to beware of ionizing radiation.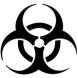** |  | 82 (39.61) |  |
| **9. CT scans (in separate rooms) do not require personal protective equipment and auxiliary protective facilities for radiological workers.** |  | 45 (21.74) |  |
| **12. In addition to regular medical examinations, personnel engaged in radiological work should also undergo examinations targeted at radiation-sensitive tissues and organs, including eye examinations and cytogenetic examinations.** |  | 173 (83.57) |  |
| **13. Personnel engaged in radiological work should also undergo skin examinations.** |  | 153 (73.91) |  |
| **14. Radiation protection measures for patients include wearing lead clothing, lead aprons, lead hats, and lead collars.** |  | 183 (88.41) |  |

Supplementary Table 2. Attitude and Practice responses among diagnostic radiographers

| **Attitude** | **Strongly Agree N(%)** | **Agree N(%)** | **Neutral N(%)** | **Disagree N(%)** | **Strongly Disagree N(%)** |
| --- | --- | --- | --- | --- | --- |
| **1. You believe that implementing radiation protection during medical radiological work is crucial.** | 168 (81.16) | 25 (12.08) | 8 (3.86) | 3 (1.45) | 3 (1.45) |
| **2. You believe that radiation protection training is necessary.** | 160 (77.29) | 34 (16.43) | 10 (4.83) | 0 | 3 (1.45) |
| **3. You believe that the current radiation protection measures are sufficiently reliable.** | 81 (39.13) | 68 (32.85) | 48 (23.19) | 7 (3.38) | 3 (1.45) |
| **4. You believe that you are overburdened with work.** | 67 (32.37) | 41 (19.81) | 77 (37.20) | 16 (7.73) | 6 (2.90) |
| **5. You believe that you have been exposed to excessive radiation at work.** | 31 (14.98) | 30 (14.49) | 81 (39.13) | 51 (24.64) | 14 (6.76) |
| **6. You believe that your health has been affected by radiation exposure from your work.** | 38 (18.36) | 37 (17.87) | 80 (38.65) | 43 (20.77) | 9 (4.35) |
| **7. You have considered changing jobs for health reasons.** | 29 (14.01) | 31 (14.98) | 81 (39.13) | 49 (23.67) | 17 (8.21) |
| **8. You are concerned about potential occupational exposure.** | 47 (22.71) | 52 (25.12) | 69 (33.33) | 28 (13.53) | 11 (5.31) |
| **9. You believe that occasionally failing to follow standard procedures due to busyness or negligence is understandable.** | 37 (17.87) | 29 (14.01) | 54 (26.09) | 60 (28.99) | 27 (13.04) |
| **Practice** | **Always N(%)** | **Often N(%)** | **Sometimes N(%)** | **Occasionally N(%)** | **Never N(%)** |
| **1.** **You undergo regular medical check-ups.** | 122 (58.94) | 26 (12.56) | 43 (20.77) | 11 (5.31) | 5 (2.42) |
| **2. You regularly undergo "occupational health checks for radiation workers."** | 141 (68.12) | 19 (9.18) | 31 (14.98) | 8 (3.86) | 8 (3.86) |
| **3. You regularly participate in radiation protection-related training.** | 127 (61.35) | 24 (11.59) | 40 (19.32) | 12 (5.80) | 4 (1.93) |
| **4. You wear a personal dosimeter at work.** | 154 (74.40) | 10 (4.83) | 24 (11.59) | 9 (4.35) | 10 (4.83) |
| **5. You always wear protective equipment as required at work.** | 118 (57.00) | 35 (16.91) | 32 (15.46) | 13 (6.28) | 9 (4.35) |
| **6. You inform patients about the potential hazards of radiation before an examination and guide them on protective measures.** | 139 (67.15) | 34 (16.43) | 20 (9.66) | 9 (4.35) | 5 (2.42) |
| **7. You pay special attention to the radiation protection of special populations (e.g., pregnant women, children).** | 180 (86.96) | 10 (4.83) | 11 (5.31) | 5 (2.42) | 1 (0.48) |

Supplementary Table 3. Correlation analysis

|  | **Knowledge** | **Attitude** | **Practice** |
| --- | --- | --- | --- |
| **Knowledge** | 1 |  |  |
| **Attitude** | 0.120 (P=0.084) | 1 |  |
| **Practice** | 0.390 (P<0.001) | 0.143 (P=0.039) | 1 |

Supplementary Table 4. Model Fit Indices for Structural Equation Model

| Indicators | Reference | Results |
| --- | --- | --- |
| RMSEA | <0.08 Good | 0.053 |
| SRMR | <0.08 Good | 0.070 |
| TLI | >0.8 Good | 0.909 |
| CFI | >0.8 Good | 0.921 |

Supplementary Table 5: Results of path analysis showing direct, indirect, and total effects between Knowledge, Attitude, and Practice domains.

| Model paths |  | Total effects | | Direct Effect | | Indirect effect | |
| --- | --- | --- | --- | --- | --- | --- | --- |
|  |  | β (95% CI) | P | β (95% CI) | P | β (95% CI) | P |
| Asum <- |  |  |  |  |  |  |  |
|  | Ksum | 0.105(-0.012,0.224) | 0.081 | 0.105(-0.012,0.224) | 0.081 |  |  |
| Psum <- |  |  |  |  |  |  |  |
|  | Asum | 0.092(-0.026,0.211) | 0.127 | 0.092(-0.026,0.211) | 0.127 |  |  |
|  | Ksum | 0.322(0.218,0.426) | <0.001 | 0.312(0.208,0.416) | <0.001 | 0.009(-0.006,0.026) | 0.25 |

Supplementary Table 6: Distribution of Knowledge, Attitude, and Practice scores by categorical classification based on Bloom's cutoff.

| Kgroup | N | Percent(%) |
| --- | --- | --- |
| <16 | 45 | 21.74 |
| [16,24) | 98 | 47.34 |
| ≥24 | 64 | 30.92 |
| Agroup |  |  |
| <25 | 39 | 18.84 |
| [25,34) | 114 | 55.07 |
| ≥34 | 54 | 26.09 |
| Pgroup |  |  |
| <28 | 47 | 22.71 |
| [28,35) | 84 | 40.58 |
| ≥35 | 76 | 36.71 |

Supplementary Table 7: Results of regression analysis showing factors associated with good Knowledge, Attitude, and Practice

|  | OR (95%CI) | P |
| --- | --- | --- |
| Regression analysis for Knowledge |  |  |
| Age (>30 vs. <30) | 2.412(1.082,5.375) | 0.031 |
| Professional title (none vs. Junior) | 0.118(0.035,0.399) | 0.001 |
| Regression analysis for Attitude |  |  |
| Professional title (none vs. Junior) | 13.95(2.701,72.07) | 0.002 |
| Age (>30 vs. <30) | 3.107(1.257,7.679) | 0.014 |
| Perceived Radiation-Related Health Issues (no vs. yes) | 3.510(1.347,9.144) | 0.010 |
| Radiation Worker Certificate (no vs. yes) | 0.130(0.026,0.638) | 0.012 |
| Average Daily Imaging Examinations (50-100 vs <50) | 0.297(0.104,0.848) | 0.023 |
| Average Daily Imaging Examinations (> 200 vs < 50) | 0.268(0.080,0.901) | 0.033 |
| Regression analysis for Practice |  |  |
| K | 1.177(1.087,1.275) | <0.001 |
| A | 1.115(1.030,1.208) | 0.007 |
| Age (>30 vs. <30) | 2.773(1.262,6.091) | 0.011 |
