# Supplementary figures and images for: Knowledge, attitude, and practice regarding radiation exposure and protection among diagnostic radiographers in radiology departments in Shanghai, China
Source: J Appl Clin Med Phys. 2026 Mar 31;27(4):e70559. doi: 10.1002/acm2.70559 (PMC13140916; doi:10.1002/acm2.70559)

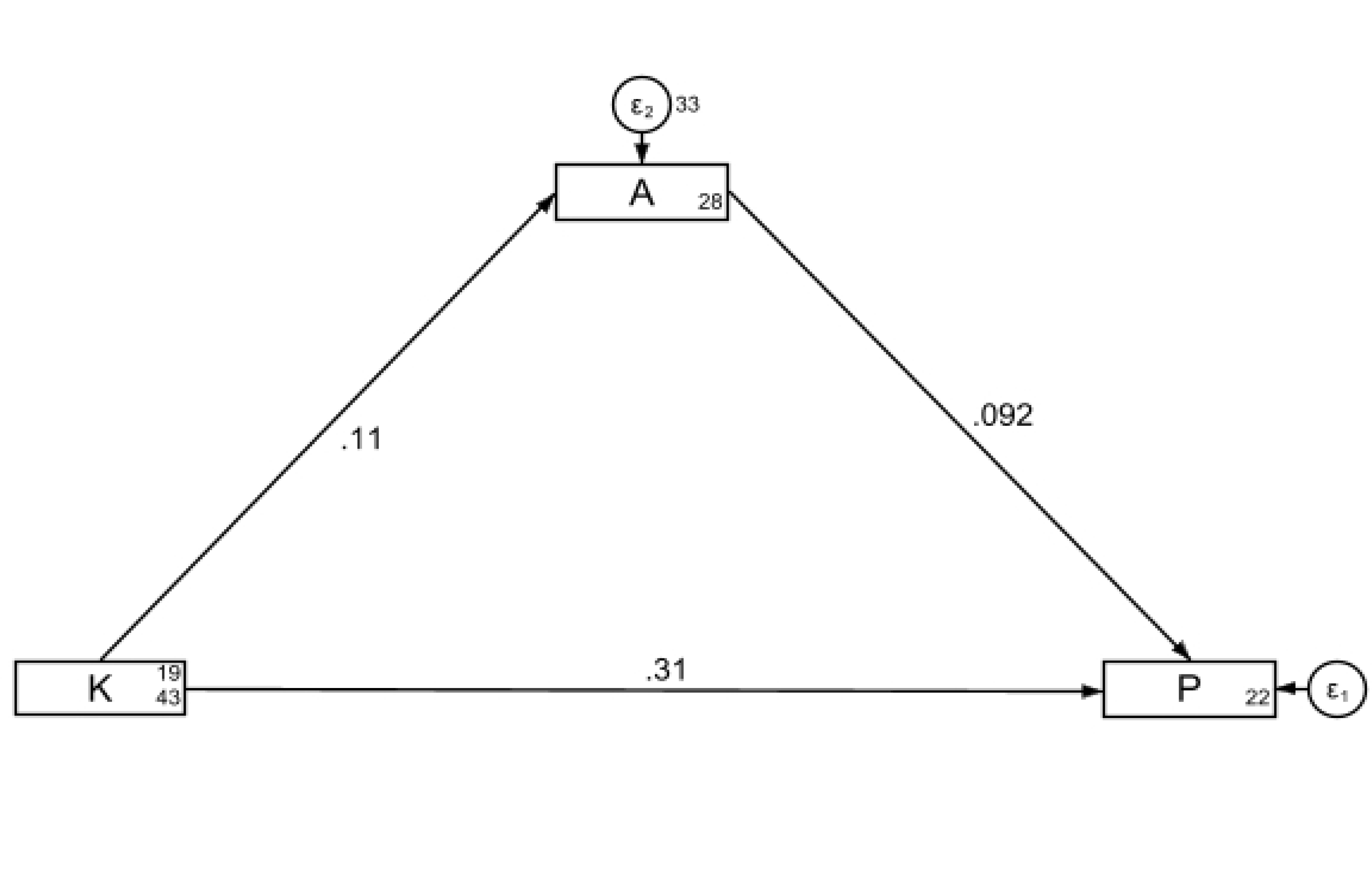

Supplement: Supplementary file 2 — Supporting File 2: acm270559‐sup‐0002‐Figure‐S01.tif. [file ACM2-27-e70559-s001.tif]
